# Evolutionary History of the GABA Transporter (GAT) Group Revealed by Marine Invertebrate GAT-1

**DOI:** 10.1371/journal.pone.0082410

**Published:** 2013-12-03

**Authors:** Azusa Kinjo, Tomoko Koito, So Kawaguchi, Koji Inoue

**Affiliations:** 1 Graduate School of Frontier Sciences, The University of Tokyo, Kashiwa, Chiba, Japan; 2 Atmosphere and Ocean Research Institute, The University of Tokyo, Kashiwa, Chiba, Japan; 3 College of Bioresource Sciences, Nihon University, Fujisawa, Kanagawa, Japan; 4 Australian Antarctic Division, Kingston, Tasmania, Australia; American University in Cairo, Egypt

## Abstract

The GABA transporter (GAT) group is one of the major subgroups in the solute career 6 (SLC6) family of transmembrane proteins. The GAT group, which has been well studied in mammals, has 6 known members, i.e., a taurine transporter (TAUT), four GABA transporters (GAT-1, -2, -3, - 4), and a creatine transporter (CT1), which have important roles in maintaining physiological homeostasis. However, the GAT group has not been extensively investigated in invertebrates; only TAUT has been reported in marine invertebrates such as bivalves and krills, and GAT-1 has been reported in several insect species and nematodes. Thus, it is unknown how transporters in the GAT group arose during the course of animal evolution. In this study, we cloned GAT-1 cDNAs from the deep-sea mussel, *Bathymodiolus septemdierum*, and the Antarctic krill, *Euphausia superba*, whose TAUT cDNA has already been cloned. To understand the evolutionary history of the GAT group, we conducted phylogenetic and synteny analyses on the GAT group transporters of vertebrates and invertebrates. Our findings suggest that transporters of the GAT group evolved through the following processes. First, GAT-1 and CT1 arose by tandem duplication of an ancestral transporter gene before the divergence of Deuterostomia and Protostomia; next, the TAUT gene arose and GAT-3 was formed by the tandem duplication of the TAUT gene; and finally, GAT-2 and GAT-4 evolved from a GAT-3 gene by chromosomal duplication in the ancestral vertebrates. Based on synteny and phylogenetic evidence, the present naming of the GAT group members does not accurately reflect the evolutionary relationships.

## Introduction

The solute carrier family 6 (SLC6) is one of the major families of transmembrane proteins, which include transporters for neurotransmitters, amino acids, and osmolytes. These transporters are classified into four groups: γ-aminobutyric acid (GABA) transporter (GAT), amino acid transporter, amino acid/orphan transporter, and monoamine transporter, based on sequence similarity and substrate specificity [1,2]. 

Concerning the GABA group, six members, comprising four subtypes of GABA transporters (GAT-1, GAT-2, GAT-3, and GAT-4 or BGT-1), taurine transporter (TAUT), and creatine transporter (CT1) have been discovered in mammals [3–7]. Although having different affinities for the substrates transported, all members can transport GABA [8], which is the major inhibitory neurotransmitter in the mammalian nervous system. Of the group members, the four GATs have the highest affinity with GABA. GAT-1 and GAT-3 participate in regulating the concentration of GABA in the brain [9]. GAT-2 is present in the brain as well as in the liver and kidney [10] and GAT-4 is able to transport betaine, which acts as an osmolyte [6]. TAUT also transports osmolytes such as taurine, hypotaurine, and β-alanine [11–14]. CT1 mainly transports creatine, one of the β-amino acids, which acts as a part of an intracellular energy buffering systems to maintain ATP levels in the heart, brain, and skeletal muscle [15,16] . 

GAT group members have been also cloned from invertebrates such as *Drosophila melanogaster*, *Manduca sexta*, and *Trichoplusia ni*, and the nematode *Caenorhabditis elegans* [17–20]. TAUT has been cloned from some marine species, including the Antarctic krill *Euphausia superba* (accession number: AB819740) and the bivalves *Crassostrea gigas*, *Mytilus galloprovincialis*, *Bathymodiolus septemdierum*, and *Bathymodiolus platifrons* [13,14,21,22]. Since all these species are protostomes, it is likely that GAT-1 and TAUT arose before the separation of protostomes and deuterostomes. However, this hypothesis cannot be proven directly because these two transporters have not been found together in any single species of deuterostome. Even in *C. elegans* and *Drosophila* spp., for which the whole genomes have been sequenced, a TAUT-like sequence has not been found. In addition, while bivalve TAUTs are functionally very similar to vertebrate TAUTs (named based on the function), their molecular-phylogenetic origin does not appear to be the same as vertebrate TAUTs [22]. Despite the importance of deep understanding of this interesting family of proteins, the evolutionary history of the GAT group remains largely unknown.

In this report, we reveal the evolutionary history of the GAT group. We cloned GAT-1 cDNA from two marine invertebrates, namely, *E. superba* and *B. septemdierum* (known to contain TAUT). To show that the separation of GAT-1 and TAUT occurred before the divergence of protostomes and deuterostomes, we collected sequence information of GAT group members from databases of various animals, including vertebrates and invertebrates, and constructed phylogenetic trees to elucidate the lineage of all 6 members of the GAT group. Finally, synteny analyses were performed using whole genome information of human, green anole, and medaka for evidence of gene duplications.

## Materials and Methods

### Sample Collection

The deep-sea mussel *Bathymodiolus septemdierum* was collected from a hydrothermal vent in Myojin Knoll at a depth of 1300 m by using a remotely operated vehicle (ROV) Hyper-Dolphin, operated by the research vessel (RV) Natsushima (Research cruise NT10-08; May 2010) of the Japan Agency for Marine-Earth Science and Technology (JAMSTEC). The Antarctic krill *E. superba* was collected off east Antarctica on February 7, 2005 (66°15′S, 74°45′E) on board the RV Aurora Australis and maintained at 0.5°C under a seasonal light regime at the Australian Antarctic Division krill experimental facility, Kingston, Tasmania [23]. Specimens were dissected, frozen with liquid nitrogen, and stored at -80°C until use. 

### Ethics Statement

Sampling of *B. septemdierum* and *E. superba* was approved by the Tokyo Metropolitan Government (permit number 22-5) and Commonwealth of Australia under Antarctic Marine Living Resources Conservation Act 1981 (permit number 00/02), respectively. All experiments were conducted according to the Guideline for Care and Use of Animals approved by the University of Tokyo committee. 

### Partial cDNAs

Total RNA was isolated from tissues of a single individual of *B. septemdierum* and *E. superba* using ISOGEN (Nippon Gene, Toyama, Japan). A double-stranded cDNA pool was synthesized from 2 μg of total RNA from *B. septemdierum* gills and *E. superba* eyes, by using a SMART cDNA Library Construction Kit according to the manufacture’s protocol (Clontech, Palo Alto, CA). ExTaq HS DNA polymerase (Takara Bio, Otsu, Japan) was used for all PCRs unless otherwise indicated. 

Touchdown PCR (TD-PCR) was carried out to amplify the partial sequences of *B. septemdierum* GAT-1 cDNA with degenerate primers GAT-1-F-3 (TGGACNGGNAARGTNGTNTA) and GAT-1-R-2 (CCYTNAYNGTRCCARAAYTG) under the following cycle conditions: predenaturation at 98°C for 1 min followed by 20 cycles of denaturation at 98°C for 10 s, primer annealing at 56.5–46.5°C (the temperature was decreased by 0.5°C every cycle) for 30 s, extension at 72°C for 60 s, and then 25 additional cycles of denaturation at 98°C for 10 s, primer annealing at 46.5°C for 30 s, extension at 72°C for 60 s, and the final extension at 72°C for 7 min. The partial sequence of *E. superba* GAT-1 cDNA was also obtained by TD-PCR using GAT-1-F-2 (AARAAYGGNGGNGGNGCNTT) and GAT-1-R-3 (CCARCANARYTTCCACCA) under the same cycle conditions used to amplify *B. septemdierum* GAT-1 cDNA, except that primer annealing temperatures for 20 cycles and 25 additional cycles were changed to 65–55°C (the temperature was decreased by 0.5°C every cycle until it reached 55°C) and 55°C, respectively. 

### The 5′-end of cDNAs

The 5′-end of GAT-1 cDNA was amplified using gene specific primers and adapter primers, 5′PCR-adapt (AAGCAGTGGTATCAACGCAGAGT) or 5′PCR-inner (AGTGGTATCAACGCAGAGTGGCCATT). However, since nonspecific PCR products were amplified with the primer combination, the following approach was used to obtain the target cDNA sequences specifically. First, a primary amplification was carried out by PCR using only a gene-specific primer to amplify 5′-end complementary strand of GAT-1 cDNAs specifically. A secondary amplification was then performed using the primary PCR products as the template with adapter and gene specific primers.

To amplify the 5′-end of *B. septemdierum* GAT-1 cDNA, primary PCR was performed using Bs-GSP-R3 (CAAACCCAGCAAAGATACTGGTACAAC) with the following cycling conditions: predenaturation at 98°C for 1 min followed by 35 cycles of denaturation at 98°C for 10 s, primer annealing at 60°C for 30 s, extension at 72°C for 2.5 min, and final extension at 72°C for 7 min. A second amplification was carried out by PCR using 5′PCR-inner and a gene-specific primer, Bs-GSP-R1 (TTCCTTCAGCTGCTCCAGGTAATGTAAC) under the following conditions; predenaturation at 98°C for 1 min followed by 20 cycles at 98°C for 10 s, 72.2°C for 2.5 min, 72°C for 60 s, and then 25 additional cycles of denaturation at 98°C for 10 s, primer annealing at 69.2°C for 2.5 min, extension at 72°C for 60 s, and the final extension at 72°C for 7 min.

To amplify the 5′-end of *E. superba* GAT-1 cDNA, a primary amplification by PCR was conducted using a gene specific primer, Es-GSP-R3 (AAAGAGCGCCCAACACAACACAATGAT), under the same cycle condition used in the primary amplification of *B. septemdierum* GAT-1 cDNA, except the primer annealing temperature (68°C instead of 60°C). A secondary amplification by PCR was performed using 5′-PCR-inner and Es-GSP-R1 (AGGAACATGGGCACCCCACAGCATAAC) under the following conditions: predenaturation at 98°C for 1 min followed by 20 cycles of denaturation at 98°C for 10 s, primer annealing at 73–67°C (the temperature was decreased by 0.3°C every cycle) for 2.5 min, extension at 72°C for 60 s, and then 25 additional cycles of denaturation at 98°C for 10 s, primer annealing at 67°C for 2.5 min, extension at 72°C for 60 s, and the final extension at 72°C for 7 min. 

### The 3′ -end of cDNAs

The 3′-end sequences of GAT-1 cDNAs were also amplified by the same approach used to amplify the 5′-end of *B. septemdierum* and *E. superba* cDNAs. For primary amplification, Bs-GSP-F1 and Es-GSP-F1 were used for the 3′-end complementary strand of *B. septemdierum* and *E. superba* GAT-1 cDNAs, respectively. The cycle conditions were the same as the primary PCR amplification of the *B. septemdierum* and *E. superba* cDNA 5′-ends described above, except for the annealing temperature (64°C instead of 60°C). A secondary amplification was carried out by TD-PCR using the primary PCR products as templates with gene-specific primer Bs-GSP-F1 (TTCACAGGTTTGGATTGATGCTGCAACT) for *B. septemdierum* GAT-1 cDNA and Es-GSP-F1 (CAAGGTATGGATGGAGGCTGTCTCACA) for *E. superba* GAT-1cDNA, with adaptor primer, CDSIII-adapt (ATTCTAGAGGCCGAGGCGGCCGACAT). The cycle conditions were the same as the secondary amplification for *B. septemdierum* and *E. superba* cDNA 5′-ends, except that the annealing temperature of each primer during the 20 cycles was set to 77–71°C for *B. septemdierum* (the temperature was decreased by 0.3°C every cycle until it reached 71°C) and 79–77°C for *E. superba* (the temperature was decreased by 0.1°C every cycle until it reached 77°C). Annealing temperatures during the 25 additional cycles were 71°C for *B. septemdierum* and 77°C for *E. superba*. 

### Whole coding region

The whole coding region was amplified from the first strand as a continuous sequence using the enzyme KOD-plus-Neo (TOYOBO, Tokyo, Japan ). To amplify *B. septemdierum* GAT-1 cDNA, gene-specific primers Bs-GSP-F5 (GGACATAACATAAAAAACAGTCATGCAG) and Bs-GSP-R5 (GGCTTTTTAATTACCTCATACAGAAGATA) were used while Es-GSP-F6 (GCACTTGGAGTGGGAAAGTCAGAGT) and Es-GSP-R8 (GAATGTTACTTTGATCCTTTTGTTGTGC) were used for *E. superba* GAT-1 cDNA. Overhanging dA was attached to the 5′and 3′-ends of each PCR product using 10×A-attachment mix (TOYOBO, Tokyo, Japan) for ligation. 

### Subcloning

All the final PCR products above were subcloned using pGEM-T Easy Vector System I (Promega, Madison, WI) with *E. coli* DH5α Competent Cells (TOYOBO, Tokyo, Japan). Positive clones were checked for inserts of predicted sizes by direct colony PCR with T7 and SP6 primers.

### Sequencing

To sequence the partial, 5′-end, and 3′-end fragments, direct colony PCR products derived from 8 to 20 colonies were used. The sequences of the entire coding regions were determined by direct sequencing of the amplified fragments. For confirmation, 5 and 8 subcloned plasmids of *B. septemdierum* and *E. superba*, respectively, were sequenced separately. 

### Transmembrane Domain Analysis

The transmembrane domains of the deduced peptide were analyzed using the SOSUI software (Tokyo University of Agriculture & Technology; http://bp.nuap.nagoya-u.ac.jp/sosui/).

### Phylogenetic analysis

Phylogenetic trees were constructed using the inferred amino acid sequences obtained from the two species and those of the GAT group members of vertebrates and invertebrates collected by BLAST search using human GAT members as queries. The accession numbers of the GAT group members are provided in Table S1. An alignment of amino acid sequences was performed using MAFFT version 6 [24], and sequences that failed to align were removed in automated fashion using trimAl v1.2 with *–gappyout* method [25]. The maximum likelihood phylogenetic analysis with 1000 bootstrap replicates was performed using RAxML (PROTCATWAG + Γ) [26]. In addition, Bayesian phylogenetic analyses were performed with MrBayes 3.1.2 [27]. Markov-chain Monte Carlo methods were used to generate posterior probabilities for each clade represented in the tree; 1× 10^6^ generations were run in four chains and sampled every 100 trees. The first 500 trees were discarded as burn-in before stabilization, and a majority rule consensus tree was constructed from subsequent trees. 

## Results

### GAT-1 cDNA cloning

**Figure 1:**
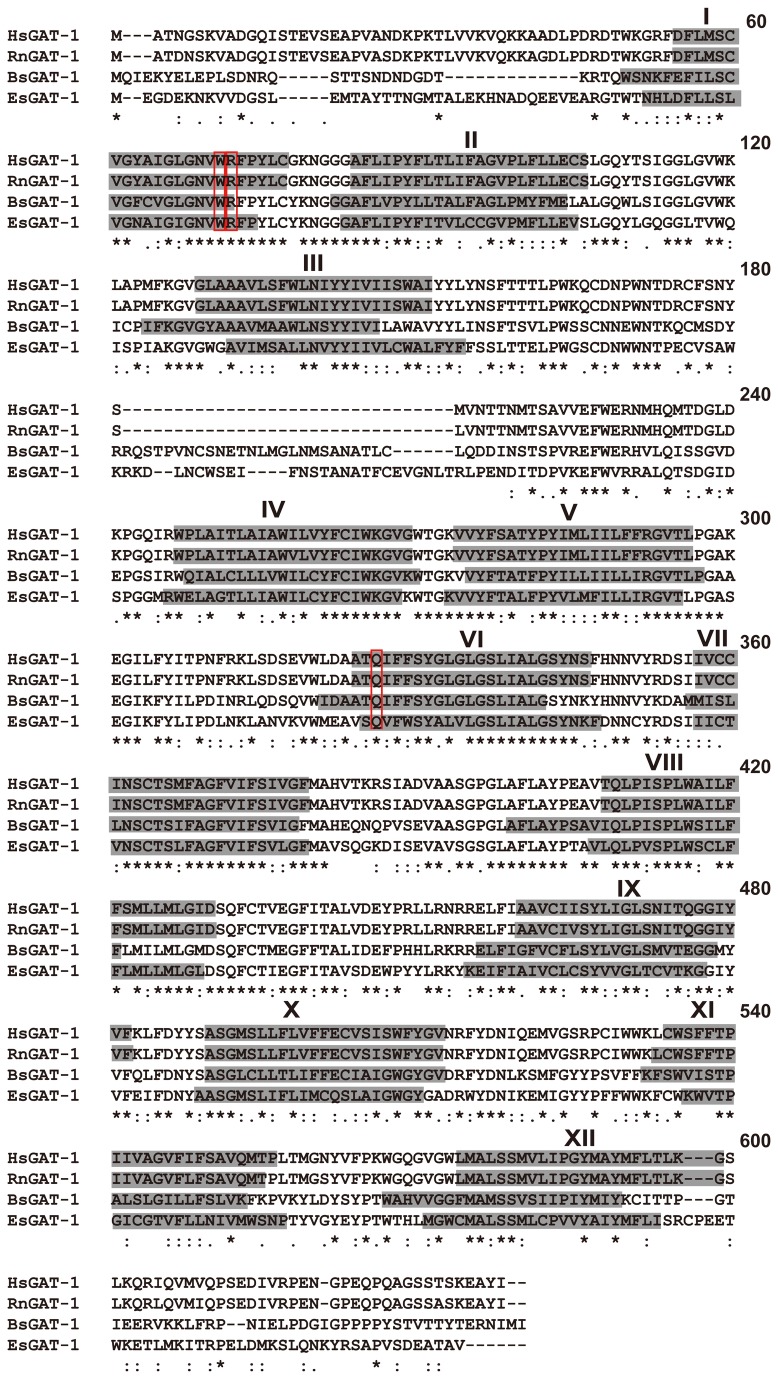
Comparison of amino acid sequences of marine invertebrate and mammalian GAT-1. Human GAT-1 (HsGAT-1), ratGAT-1 (RnGAT-1), *B. septemdierum* GAT-1 (BsGAT1), and *E. superba* GAT-1 (EsGAT1) were compared. Identical amino acids are indicated by asterisks; conservative substitutions are indicated by a single dot; putative transmembrane domains (I-XII) are shaded; red boxes indicate position of amino acids known to be important for the functions of RnGAT-1.

The cDNA sequence obtained from *B. septemdierum* contained 1953 bp and open reading frames of 611 amino acid residues (accession number: AB819738); the cDNA sequence from *E. superba* contained 2034 bp and open reading frames of 621 amino acid residues (accession number: AB819739) (Figure 1). Sequence polymorphism between the two haploid chromosomes was not found in *B. septemdierum*. The *E. superba* sequence contained a silent nucleotide polymorphism in the open reading frame (A/G at 255th nucleotide of the deposited sequence). According to the SOSUI transmembrane domain analysis, both deduced amino acid sequences contain putatively 12 transmembrane domains with a large extracellular loop between transmembrane domains 3 and 4, which is commonly observed in many members of the SLC6 [2]. Furthermore, the number of amino acid residues was close to those of known GAT group members in various animals. Both sequences displayed the highest degree of similarity with the GAT-1s from several insect species such as *D. melanogaster* (vs. *B. septemdierum*, 60%; *E. superba*, 58%), *T. ni* (vs. *B. septemdierum*, 57%; *E. superba*, 56 %), and *M. sexa* (vs. *B. septemdierum*, 56%; *E. superba*; 56%). In addition, comparisons of the amino acid sequences with human GATs showed that they have the greatest homology to human GAT-1 (vs. *B. septemdierum*, 58.2%; *E. superba*; 52.8%). Moreover, the positions of the amino acid residues (Trp^68^, Arg^69^, and Gln^291^) important for the transport functions of rat GAT-1 [28–30] were conserved after alignment in both sequences from *B. septemdierum* and *E. superba* (Figure 1). 

### Molecular Phylogenetic Analyses

**Figure 2:**
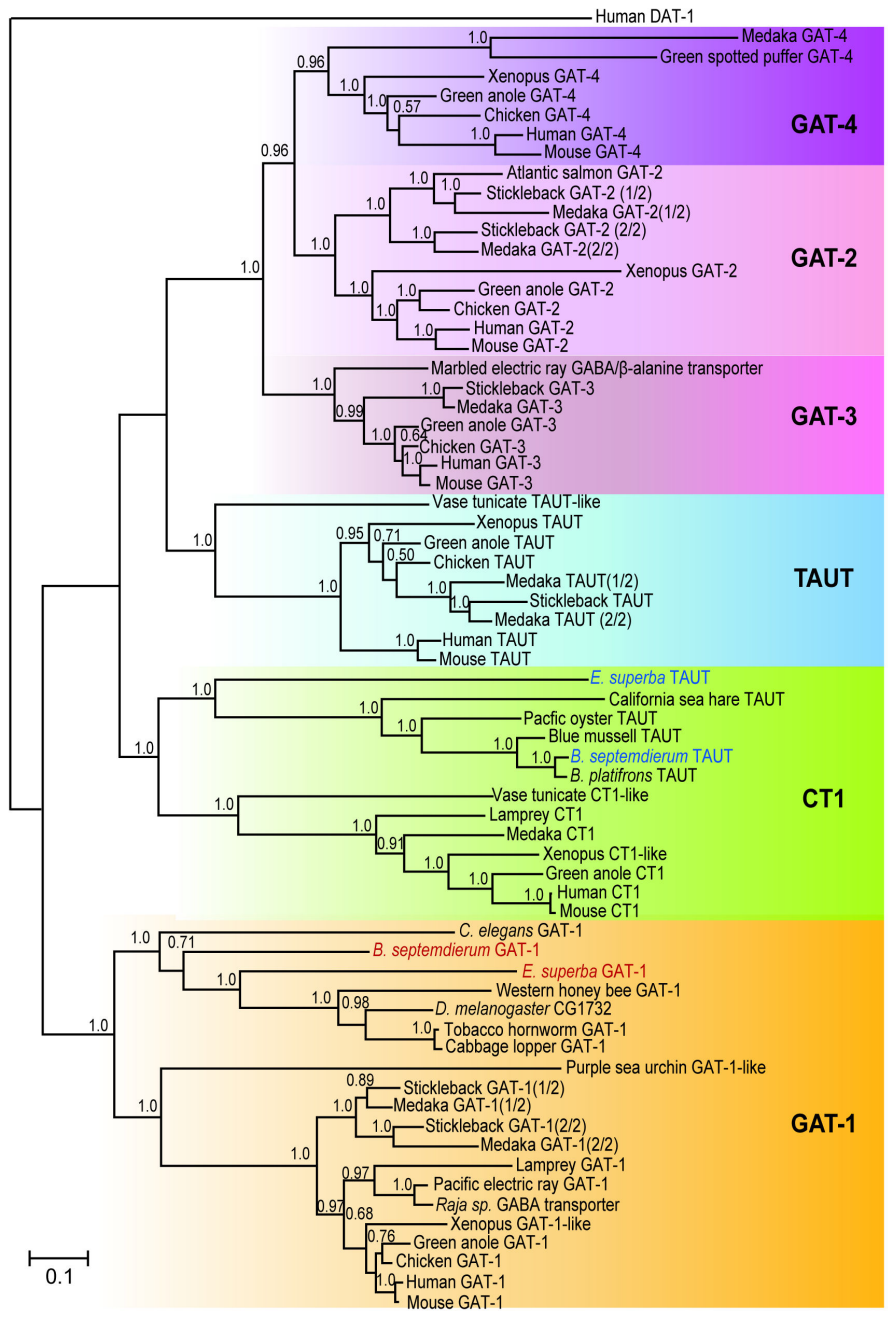
Molecular phylogenetic tree of the GAT group members of vertebrates and invertebrates constructed by Bayesian method. The posterior probabilities are shown on the top left part of a branch. The scale bar represents a phylogenetic distance of 0.1 substitutions per site. *B. septemdierum* GAT-1 (BsGAT1) and *E. superba* GAT-1 (EsGAT1) are shown in red and their TAUTs are shown in blue.

In order to confirm whether the obtained sequences from *B. septemdierum* and *E. superba* are included phylogenetically in a clade with mammalian GAT-1s and to obtain a clue in understanding how GAT members have evolved, phylogenetic trees were constructed by both Bayesian (Figure 2) and maximum likelihood (Figure S1) methods. Both trees showed very similar branching pattern of the six GAT members. The sequences obtained in this study formed a clade with GAT-1s of other organisms, indicating that they are orthologs of vertebrate GAT-1. Consequently, we named the *B. septemdierum* and *E. superba* sequences as BsGAT-1 and EsGAT-1, respectively. The GAT-1 clade is monophyletic, and the topology of the tree within the clade agreed with the phylogenetic relationship of the considered species

Although the bivalve TAUTs have been considered to be the orthologs of vertebrate TAUT according to their functional similarity, they formed a clade with CT1s with strong bootstrap value support. This finding suggests that bivalve TAUTs are actually orthologs of vertebral CT1s. 

GAT-2, GAT-3, and GAT-4 were more closely related phylogenetically to TAUT and CT1 than to GAT-1, regardless of their functional similarities to GAT-1. 

The phylogenetic trees also suggested the existence of GAT members in the tunicate *Ciona intestinalis*. Two unidentified amino acid sequences of *C. intestinalis* detected by BLAST search formed clades with CT1s and TAUTs, respectively. 

### Synteny analysis

**Figure 3:**
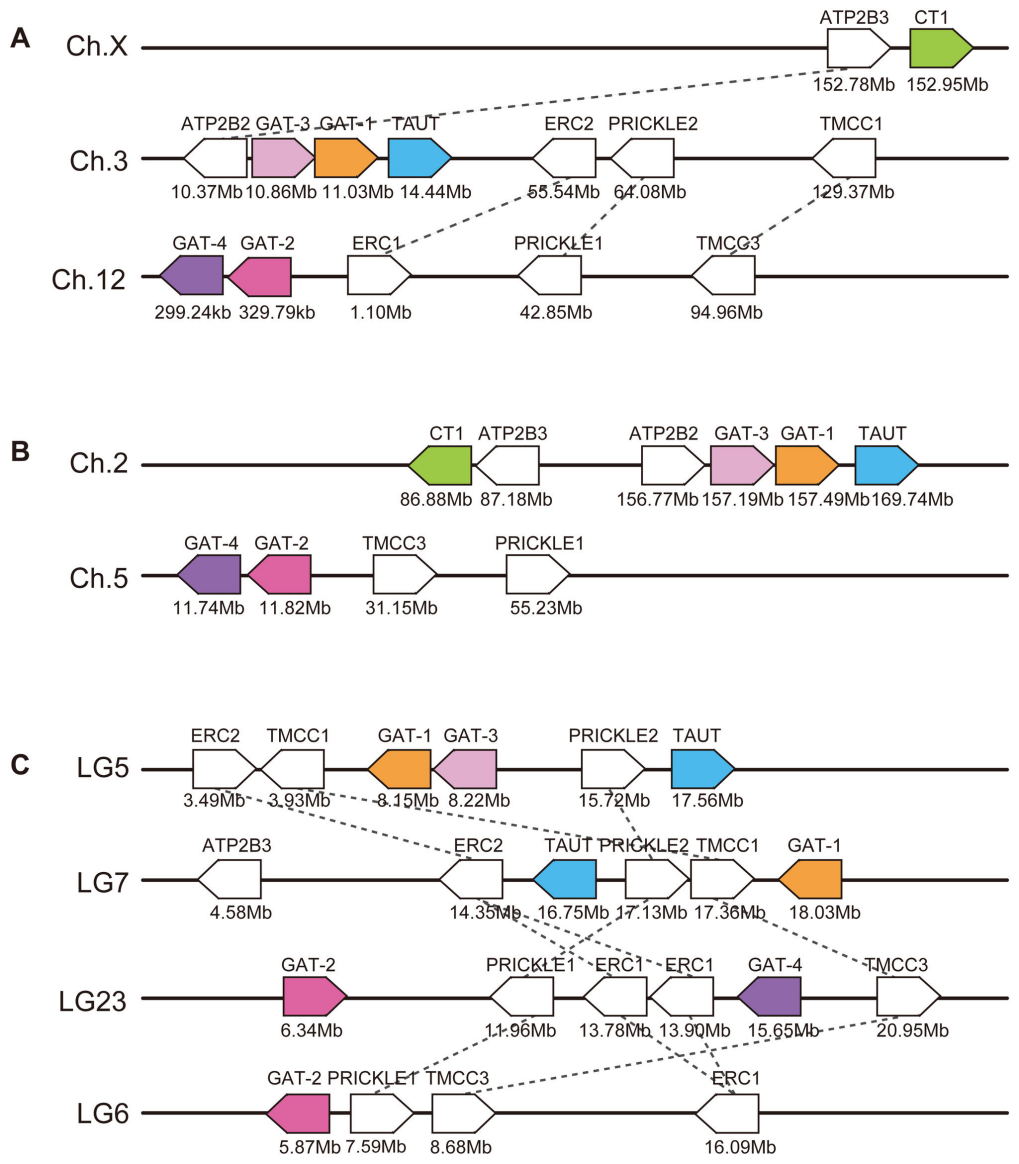
Localization of GAT group member genes on chromosomes of vertebrates. (A) human, (B) green anole, (C) medaka. Ch, chromosome; LG, linkage group.

Localization of the GAT group member genes on the chromosomes of three vertebrate species, namely, medaka, green anole, and human, were compared. The GAT-1, GAT-3, and TAUT genes were located in tandem on the same chromosome in medaka (LG5), green anole (chromosome2), and human (chromosome 3) (Figure 3). Similarly, the genes for GAT-2 and GAT-4 genes were located in tandem on the same chromosome in medaka (LG23), green anole (chromosome 5), and human (chromosome 12). In addition, another GAT-2 gene was found on LG6 of medaka as well as additional copies of GAT-1 and TAUT genes on LG7. The CT1 gene was located on human chromosome X. Green anole had a CT1 gene on the same chromosome that contained GAT-1/GAT-3/TAUT genes (chromosome 2), but was distant from the three genes. A CT1-like gene was found in a genome contig of medaka although the location of the contig on the chromosome map has not been determined. 

Some common paralogous genes of ELKS/RAB6-interacting/CAST family members (ERC1 and ERC2), PRICKLE (PRICKLE1 and PRICKLE2), transmembrane and coiled-coil domain family members (TMCC1 and TMCC3), plasma membrane calcium-transporting ATPases (ATP2B2 and ATP2B3) were found around genes of GAT members on the chromosomes of the three species (Figure 3). 

## Discussion

Cloning of GAT-1s from *B. septemdierum* and *E. superba* offers the first clues for the elucidation of the GAT group evolutionary history. This is the first study to show that both GAT-1 and CT1 (bivalve TAUT) genes are present in a single protostome species, verifying that they appeared before the separation of protostomes and deuterostomes. The phylogenetic analyses located the clade of GAT-1 at the most basal position. Since GAT-1 has been conserved in both invertebrates and vertebrates, it may have ubiquitous and fundamental functions in animals. In mammals and nematodes, GAT-1 transports GABA, one of the major inhibitory neurotransmitters, and is involved in the termination of GABA signaling at the synapse [31,32]. Further studies are needed to confirm whether GAT-1s of *B. septemdierum* and *E. superba* have the similar roles as those of other organisms. 

The second transporter that diversified among the GAT members was CT1. Although bivalve TAUTs exhibit high affinity for taurine, phylogenetic analyses revealed that they belong to the same cluster as CT1s of other organisms, indicating they are actually an ortholog of CT1. The CT1 gene has not been cloned in protostomes thus far, and the vertebrate-type TAUT genes have not been found in protostomes. However, creatine, a major substrate of CT1 is present in protostomes and actively transported from seawater [33]. Possibly, just one protein plays a role in transporting both taurine and creatine in protostomes. Further studies are needed to confirm whether bivalve CT1 is able to transport creatine. 

After CT1 diversified, the TAUT (the vertebrate-type) gene diversified. Since TAUT-like genes are found in the tunicate, it appears that TAUT existed at an early stage of the deuterostome　lineage. Moreover, the phylogenetic trees imply that the TAUT gene (the vertebrate-type) possibly appeared before the separation of protostomes and deuterostomes. If this is the case, then the TAUT (vertebrate-type) gene may have been lost in the protostome lineage since it is not currently available in the genome databases of protostomes such as *D. melanogaster*, *C. intestinalis, Lottia gigantea*, and *Pinctada fucata martensii*.

The GAT-3 gene was likely generated by the tandem duplication of the ancestral TAUT gene since the GAT-3 gene exists close to the TAUT gene on the same chromosome. However, GAT-3 is functionally similar to GAT-1, rather than TAUT, i.e., it has the highest affinity to GABA, and functions in neurons [8]. GAT-3 has been found only in vertebrates, and thus it may have arisen in the vertebrate lineage. The appearance of the GAT-3 gene may be linked to the development of complex nervous systems in vertebrates. Recently, differences in sub-cellular localization in neurons were reported for GAT-1 and GAT-3 [34].

Synteny analyses showed that common paralogous genes are present on the chromosome containing the GAT-2 and GAT-4 genes and on the chromosome containing GAT-1, GAT-3, and TAUT genes (Figure 3). This suggests that the chromosome containing the GAT-2 and GAT-4 genes was created by duplication of the chromosome containing the GAT-1, GAT-3, TAUT genes. Since medaka has both GAT-2 and GAT-4 genes, it is evident that the chromosome duplication occurred prior to the divergence of the ray-finned and lobe-finned fish lineages. Although it is known that whole genome duplications occurred twice (known as 1R and 2R) during vertebrate evolution [35,36], it is difficult to determine the exact time of the duplication event because neither GAT-2 nor GAT-4 genes of agnathans and cartilaginous fishes has been detected in the currently available genome databases. Further investigation of the genomes of primitive vertebrates is needed to answer this question.

Our phylogenetic tree indicates that both GAT-2 and GAT-4 are more similar to GAT-3 than to TAUT and GAT-1. This result suggests that both GAT-2 and GAT-4 were derived from a copy of the GAT-3 gene, not from the GAT-1 or TAUT gene; i.e., the copy of the GAT-3 gene underwent tandem duplication, and generated the ancestral GAT-2 or GAT-4 gene. In addition, it is likely that the two duplicated sets of chromosomes bearing the GAT group genes (Figure 3C) detected in medaka were generated by a third round of whole genome duplication (3R) that occurred in the teleost fish lineage [37,38]. Although genes of other GAT members may also have duplicated, we speculate that they may have been lost because two sets of the genes were not found in the medaka genome.

**Figure 4:**
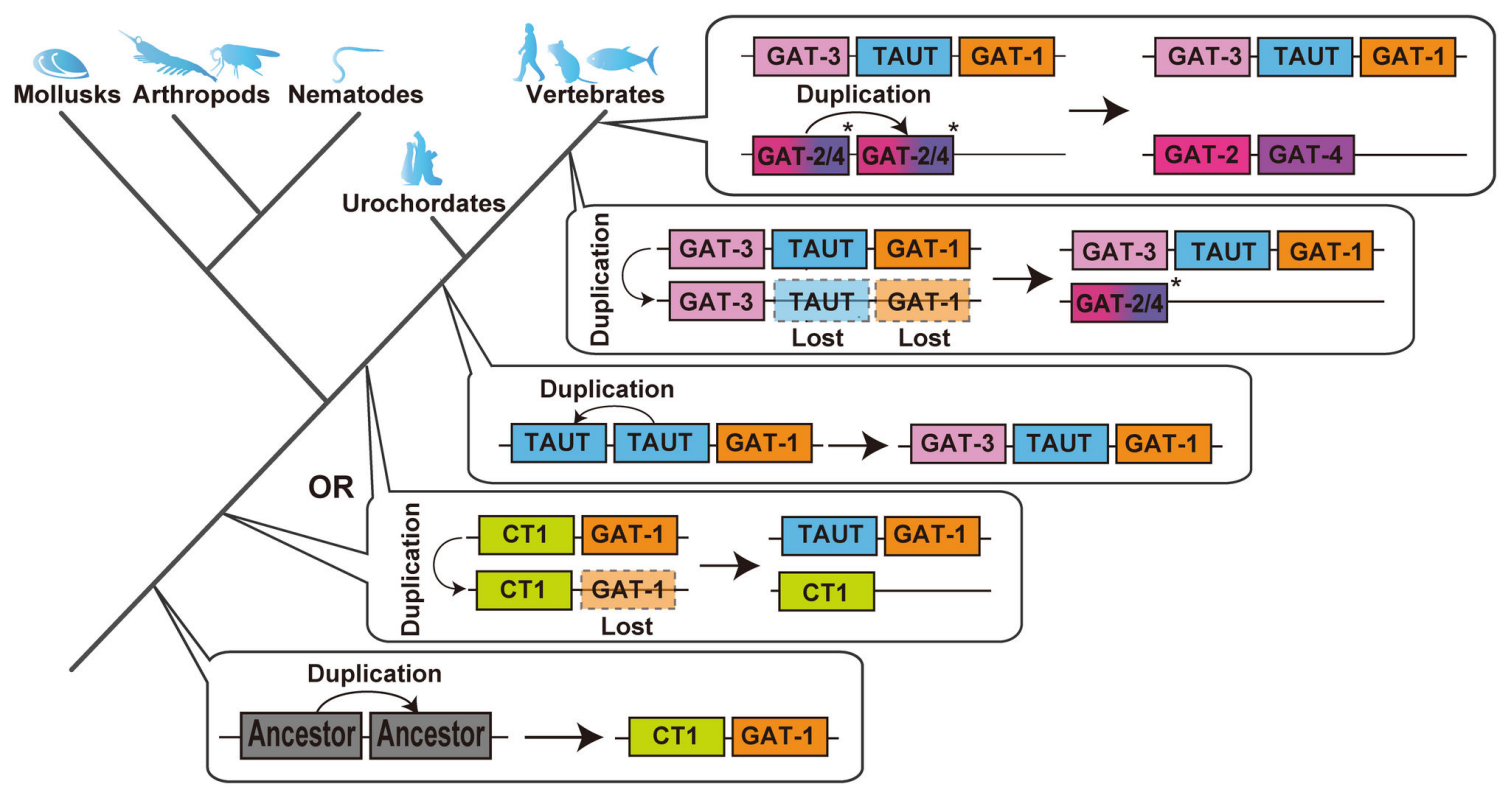
Proposed molecular evolutionary history of GAT group member genes. * Since which of GAT-2 and GAT-4 was created first is unclear, their ancestor is shown as GAT-2/4.

Based on the findings obtained in this study, we proposed the following evolutionary history of the GAT group (Figure 4). First, duplication of a common ancestral transporter gene occurred before the separation of protostomes and deuterostomes, resulting in diversification of GAT-1 and CT1. Next, TAUT was generated by duplication of CT1 before the separation of the protostomes and deuterostomes, or only in the deuterostome lineage. If the latter is the case, then protostome CT1 may play a role in the transportation of both creatine and taurine because no conventional TAUT has been found in protostomes. TAUT experienced further duplication, which generated GAT-3 prior to the common ancestral vertebrates. Finally, the common ancestor of GAT-2 and GAT-4 was generated by chromosome duplication of GAT-3, which occurred at 1R or 2R, followed by the tandem duplication of the common ancestral gene that generated GAT-2 and GAT-4. With the 3R event in the teleost lineage, GAT-1, GAT-2 and TAUT genes were further duplicated. 

According to the evolutionary history proposed here, GAT-2, -3, or -4 were not derived directly from GAT-1. In addition, we found that the invertebrate transporter that has been called TAUT is actually in the lineage of vertebrate CT1. The names of the GAT group members do not accurately reflect the phylogenetic relationships; thus, it may be necessary to reconsider the naming of the transporters in the GAT group. The molecular lineage revealed in this study also provides clues for functional studies. In the future, we will examine the phylogenetic relationships proposed in this study from the functional viewpoint. 

## Supporting Information

Figure S1 **Molecular phylogenetic tree of the GAT group members of vertebrates and invertebrates constructed by ML method.** Bootstrap values are shown on the top left part of a branch. The scale bar represents a phylogenetic distance of 0.1 substitutions per site.
(TIF)

Table S1 **Accession numbers of amino acide sequences of the GAT group members used in phylogenetic analyses.**
(XLSX)
