# Supplementary figures and images for: Evolutionary History of the GABA Transporter (GAT) Group Revealed by Marine Invertebrate GAT-1
Source: PLoS One. 2013 Dec 3;8(12):e82410. doi: 10.1371/journal.pone.0082410 (PMC3849432; doi:10.1371/journal.pone.0082410)

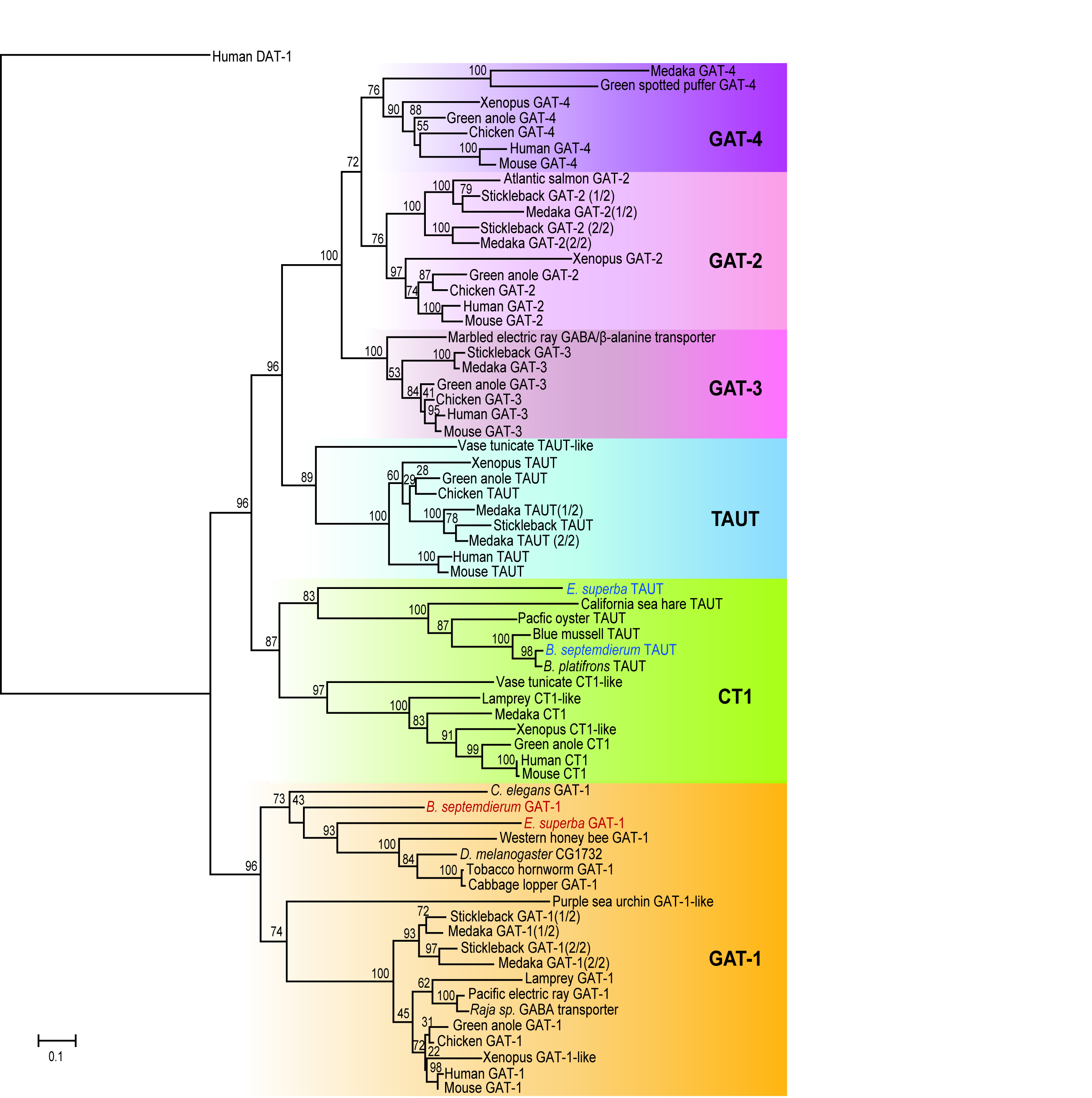

Supplement: Figure S1 — Molecular phylogenetic tree of the GAT group members of vertebrates and invertebrates constructed by ML method. Bootstrap values are shown on the top left part of a branch. The scale bar represents a phylogenetic distance of 0.1 substitutions per site. (TIF) [file pone.0082410.s001.tif]
